# A Commentary on Commercial Genetic Testing and the Future of the Genetic Counseling Profession

**DOI:** 10.1007/s10897-018-0244-6

**Published:** 2018-03-09

**Authors:** Nicholas D. Wolff, Jon A. Wolff

**Affiliations:** 1 Genetic Support Foundation, 1800 Cooper Point Road SW #14, Olympia, WA 98502 USA; 2 Department of Pediatrics, University of Wisconsin School of Medicine and Public Health, Madison, WI USA

**Keywords:** Genetic testing, Genetic counseling, Medical genetics, Clinical laboratories

## Abstract

Commercial genetic testing laboratories are increasingly employing genetic counselors. As a result, the role of these or many genetic counselors is shifting from primarily direct patient counseling in clinics and hospitals to more laboratory-centered activities that involve case coordination, customer liaison, variant classification, marketing, and sales. Given the importance of these commercial entities to the genetic counseling profession, this commentary examines the current financial situation of four publicly traded, genetic testing companies. It also explores how the various roles of genetic counselors are likely to be affected by the financial pressures these companies face.

## Introduction

Commercial genetic testing laboratories are playing an increasing role in the development and delivery of genetic services, influencing the protocols and practices of medical genetics in the USA (Strom 2012). They are spurring the introduction of new genetic services and tests such as the use of non-invasive prenatal screening and cancer genetic testing. The increased influence of commercial genetic testing laboratories has caused many changes in the medical genetics work force and genetic counselors, in particular. The role of genetic counselors is shifting from primarily, direct patient counseling in clinics and hospitals to more laboratory-centered activities that involve case coordination, customer liaison, variant classification, marketing, and sales (Waltman et al. 2016). For example, in Washington State, the percentage of genetic counselors working in a clinical setting decreased from 60% in 2011 to 42% in 2015. Genetic counselors employed by commercial laboratories increased from 21% in 2011 to 42% in 2015 (Mackison and Stoll 2016). In a 2016 Professional Status Survey conducted by the National Society Genetic Counselors (NSGC), 1524 respondents counsel patients while 508 respondents do not (NSGC 2016). Approximately 50% of the respondents who do not counsel patients work for commercial laboratories (*n* = 252), while only 6% of the respondents who do counsel patients reported that they work for commercial laboratories (*n* = 97).

This article examines the financial health of such companies because this will affect their long-term influence on the practice of medical genetics and the employment of genetic counselors. It is primarily focused on publicly traded companies that offer genetic testing with significant genetic counseling implications. This includes not only genetic testing for hereditary cancer susceptibility (e.g., *BRCA1* and *BRCA2*) but also prenatal genetic testing such as cell-free DNA screening (NIPS; non-invasive prenatal screening), carrier screening, and microarray for prenatal diagnosis. It is important to note that many commercial genetic testing laboratories, including those we are reviewing, also have genetic-based products for somatic oncology (i.e., detecting genetic changes in cancers that are not necessarily germ-line derived). While such tests have potential diagnostic and therapeutic implications, they generally have less direct genetic counseling implications. As an example of tests for which genetic counseling is important are Myriad Genetics BRACAnalysis (legacy BRCA1 and BRCA2 analysis) or the myRisk®, 28-gene panel for evaluating a patient’s risk for developing various hereditary cancers. In contrast, Myriad’s Prolaris® test analyzes transcripts in prostate cancer for prognostic purposes and is not expected to have significant implications for genetic counseling.

While dozens of commercial laboratories perform genetic testing, only a handful of them are publicly traded and have easily accessible financial information. Given that this information is necessary for an analysis of a company’s financial status, we will focus on publicly traded commercial genetic testing laboratories. We assume that the financial landscape should be similar for both, except for the added cost of maintaining a publicly traded company (at least $1 million/year). Some of the publicly traded companies that offer genetic testing are part of larger companies that perform many types of laboratory testing (e.g., Quest and LabCorp) with genetic testing being a smaller percentage of total sales. We choose not to look at these companies because their multifaceted activities limit our ability to delineate the financial aspects for their laboratories’ genetic tests. Eliminating companies that do not match our criteria, we are left with four publicly traded, commercial laboratories whose primary focus is genetic testing—Myriad Genetics, Natera, Invitae, and CombiMatrix.

## Background: Typical Trajectory of Start-up Companies

**Fig. 1:**
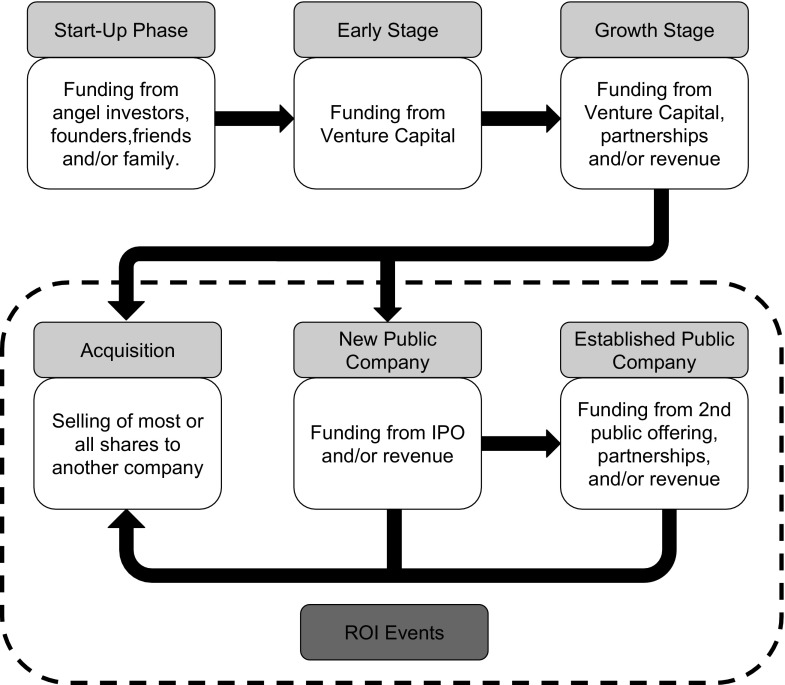
Typical trajectory of start-up companies. Abbreviations: ROI, return-on-investment; IPO, initial public offering

Before analyzing these four companies, it would be useful to review the typical trajectory of start-up companies in relation to their funding sources (Fig. 1). This will give useful insight to the expected paths of these companies. During a company’s start-up period, they use funds from angel investors (wealthy individuals who invest their own monies), founders, friends, and family to get the company created. After the company reaches some milestone such as a proof-of-concept demonstration, an early-stage company secures funds from venture capital (a type of private equity) to expand operations or research and development (R&D). Companies in later stages often get additional monies from venture capital, partnerships, or revenue to expand their activities to reach profitability.

When a company becomes public, it typically tenders an initial public offering (IPO) which is the first time the company’s stock is offered to the public. Proceeds from the IPO are used to support the company’s growth. After a company becomes public, it may not be able to generate sufficient revenue to fuel its expansion, so it raises additional monies by having another public offering (2nd PO) or forming partnership with other companies.

Typically, after about 5 years, the company’s early investors expect to make money off their investment in what is called a return-on-investment (ROI) event. This often happens after a company becomes public and has an IPO because it is easier for publicly traded shares to be sold. Another ROI event is when either a private or public company is bought by another company. Finally, if a company becomes profitable, it may provide a ROI by offering dividends to its shareholders. Other shareholders such as the founders and employees can also benefit financially from these ROI events. This makes a ROI event a key objective for start-up companies.

Our four public companies under consideration—Myriad Genetics, CombiMatrix, Natera, and Invitae—went through these growth routes to have IPOs in 1995, 2007, 2015, and 2015, respectively. It is also of interest to consider the trajectories of some genetic testing companies that have been acquired. Sequenom, one of the pioneering companies in non-invasive prenatal screening, had their IPO in 2000 but was bought in 2015 by Laboratory Corporation of America (Lab Corp), a large and diversified testing company. Ariosa, another company specializing in non-invasive prenatal screening, never went public but was bought as a private company by Roche in 2015. GeneDx, a gene testing company founded in 2000, also never went public and was bought in 2006 by another large, general laboratory testing company, Bio-Reference Laboratories, which was in turn bought by Opko Health in 2015. Sequenom, GeneDx, and Ariosa continue to operate as semi-independent subsidiaries. In contrast, Signature Genomics, formed in 2003, was a leader in microarray-based testing, and was bought as a private company in 2010 by PerkinElmer, but subsequently shut its operations in 2014. During the review process of this paper, it was announced that CombiMatrix would essentially be acquired by Invitae via a merger process.

## A Current Financial Picture of Hereditary Testing

A comprehensive assessment of the publicly traded, genetic testing companies can be obtained from their annual 10-K reports. Public companies are required to file these yearly with the U.S. Securities and Exchange Commission (SEC) and must include an extensive tally of financial information. Table 1 summarizes the 10-K reports profits and loss statement for the fiscal year of 2016 pertaining to the four companies under consideration.

**Table 1:** 2016 revenue and losses for four publicly traded, genomic-testing companies ($ in millions)

	Myriad	Invitate	Natera	CombiMatrix
Revenue^a^	$753.8	$25.1	$217.1	$12.9
Expenses				
Cost of revenue^b^	$157.3	$27.9	$135.6	$5.8
Research and development	$70.6	$44.6	$41.9	$0.5
Selling and marketing	$359.1	$28.6	$136.1	$4.6
General and administrative	Merged with selling and marketing	$24.1	Merged with selling and marketing	$6.0
Total expenses	$429.7	$125.2	$313.6	$17.0
Operating income (loss)^c^	$166.8	($100.2)	($96.5)	($4.1)
Net income (loss)^d^	$125.3	($100.3)	($95.1)	($4.1)

Source for these numbers are from the companies’ SEC 10-K filings

^a^Revenue from testing, licensing, pharmaceutical services, and other sources

^b^The costs for receiving test samples, performing tests, reporting test results, and other activities directly related to an individual test

^c^Obtaining by subtracting “total expenses” from “revenue”

^d^“Operating income” plus other income (expenses), taxes, and interest expense

It appears that Myriad is the only company, of our four selected, that was profitable in 2016. Of its total revenue of $723 million, the bulk of their revenue continues to result from their hereditary cancer testing franchise ($632 million). The other three companies, Invitae, Natera, and CombiMatrix, all had substantial net losses in 2016 (Table 1).

The financial challenges of these companies have not escaped the attention of investment analysts and “Wall Street” and their stock prices and market capitalizations have all decreased in recent years. A company’s market capitalization is the total worth of a company and is calculated by multiplying its stock price (i.e., the cost of one share) by the total number of outstanding shares. Myriad’s market capitalization decreased from approximately $2.5 billion at the beginning of 2015 to approximately $1.2 billion at the beginning of 2017. Similarly, Natera’s market capitalization decreased from approximately $1.1 billion soon after it went public on 2 July 2015 to approximately $620 million at the beginning of 2017. Invitae’s market capitalization decreased from approximately $550 million soon after it went public on 12 February 2015 to approximately $300 million at the beginning of 2017. CombiMatrix market capitalization has been about $15 million over the last several years despite several swings up and down.

## The Plans of the Genetic Testing Companies

In the reports filed with the SEC, each of these companies acknowledge their challenging financial situations and each company plans to respond to these challenges in various ways. Their 10-K annual reports detail their strategies for doing such.

The business model by which Myriad Genetics achieved its success through its cancer gene testing franchise is no longer as feasible given the 2012 *Mayo v. Prometheus* and 2013 *Molecular Pathology v. Myriad Genetics* Supreme Court decisions (Cook-Deegan and Niehaus 2014). Given the intense competition that Myriad has experienced since the patent ruling, the company plans to increase its emphasis in the development of non-hereditary tests (Myriad 2016). They hope to do this largely by acquiring companies that sell such tests. Myriad also hopes to increase foreign sales of both their hereditary and non-hereditary testing franchises.

Invitae aims to increase its market share for hereditary testing for cancer and other conditions by decreasing its operating costs to run tests, increasing its volume of tests performed and enhancing their customer experience (Invitae 2016). The company’s recent announcement about the expansion of their “proactive genetic testing program” also indicates that they plan to expand their test sales through targeting the healthy portion of the population (Invitae | Patients Home n.d.). Invitae’s 10-K report indicates that they expect to reach profitability in 2018. Invitae is also developing processes and technologies to manage genetic information over the course of a disease and over the lifetime of a patient, in what they term “genome management.” Invitae anticipates that with the cost of sequencing continuing to decrease, genetic information analysis and interpretation will play a larger role in medicine by 2020. In accordance, Invitae now calls itself a “genetic information company” instead of a genetic testing company. With this in mind, they are investing in building “genome networks” (their internal term) to manage genetic information among many patients to advance the understanding and treatment of diseases. To achieve this objective, they recently purchased AltaVoice, a platform for collecting and coordinating large sets of data from patients and clinicians through patient advocacy groups. Some current and former Invitae principals are also involved in a new company, Genome Medical, whose mission is to provide genetic counseling in support of genetic testing.

CombiMatrix is hoping to continue to capitalize on their main expertise in the use of microarrays to detect chromosomal abnormalities (CombiMatrix 2016). It intends to become the pre-eminent diagnostic services laboratory for in vitro fertilization (IVF), prenatal diagnostics, miscarriage analysis, and pediatric development disorders. They seek to increase their market share in this area by a direct-to-physician sales effort. CombiMatrix also hopes to improve its reimbursement processes and rates.

Natera also seeks to compete in the prenatal testing market, with its Panoroma® cell-free DNA screen for Down syndrome and other chromosome conditions and Horizon®, their expanded carrier screening panel (Natera 2016). Natera also aims to improve its financial situation by enhancing the customer experience, reducing the cost of testing, and increasing market share. Interestingly, it is licensing its computer-based algorithms and processes for analyzing cell-free DNA to other laboratories. The company will be launching its new Evercord® cord blood and tissue banking service in 2017. In addition, they plan to leverage its expertise in cell-free DNA analysis to develop tests for the early detection and diagnosis of cancer, e.g., liquid biopsy.

## Other Factors Affecting Genetic Testing

There are several, more universal, factors that will affect the financial health of these companies. First, market size for genetic testing is expected to grow substantially in the coming years. Some experts are recommending universal BRCA gene screening (Gabai-Kapara et al. 2014). This growth of the market will come with an increase in competition in the marketplace. There are currently more than 50 labs offering genetic testing for mutations in BRCA1 and BRCA2, and as the market size for these tests continues to expand, the competition with new labs will correspondingly continue to increase (Concert Genetics 2016).

Reimbursement for genetic testing is another key element, and these testing laboratories are approaching billing in innovative ways to navigate the maze of regulations and practices that govern reimbursements. Myriad Genetics, which has been operating for many years and is “in-network” with most insurers, still primarily seeks reimbursement through traditional means of billing with both commercial and government insurances. Natera and Invitae, being newer laboratories, have worked to establish network contracts with insurance providers and financial assistance programs for out-of-network patients. They both also have significantly reduced prices for patients that elect to pay directly. As an example, Invitae charges $1500 for gene panel tests when reimbursed from insurance but only $475 when patients pay directly. Another model for re-imbursement is to avoid insurance re-imbursement all together. This is the primary reimbursement model for Color Genomics, a privately owned genetic testing company. It offers a cancer panel for only $250, a cost sufficiently low that many patients are able to pay out-of-pocket without the requirement of reimbursement from insurance companies (although recently Color Genomics is enabling third-party payment). Five companies including Natera, recently formed the Coalition for Access to Prenatal Screening (CAPS) to “encourage appropriate legislative measures and reimbursement coverage policy changes” for prenatal screening using cell-free DNA (cfDNA).

The role of health professionals in the testing process is also an issue being experimented with by genetic testing companies. There is a continuum of approaches from companies for how much genetic testing should be integrated into the broader healthcare system. While some tests are only available through health professionals, an approach pioneered by 23andMe is to offer direct-to-consumer tests that minimize involvement of health professionals. More companies, such as Natera, Invitae, and Myriad are offering a mixed approach, requiring a healthcare provider to order tests but also advertising directly to patients. Companies including Color Genomics take an approach that has been termed “physician-mediated” direct-to-consumer testing, in which the testing is requested and purchased directly by the patient, and the order is authorized by a physician that is contracted with the laboratory. How companies approach this issue will likely continue to evolve as the repercussions of recent FDA policies get integrated into the marketplace (Annas and Elias 2014). Recently, the FDA indicated that it will allow direct-to-consumer genetic testing for certain health indications (FDA 2017).

## One Genetic Test, Many Analyses, and Interpretations: a Possible Future

Genetic tests have evolved from single-gene tests to multi-gene disease panels as the cost for sequencing has steadily fallen. As these sequencing costs continue to decrease, it will soon be cost-effective to perform whole exome sequencing (WES) or whole genome sequencing (WGS) instead of multi-gene panels. Illumina has announced a new sequencing machine that will be able to do a complete genome for $100 in the next several years (Illumina 2017). Companies such as Helix, Genos, and Veritas are developing new business models centered around providing WES or WGS inexpensively to patients.

Conceivably, the cost of genetic testing will be low enough that everyone, or at least the majority of people, will have their genome sequenced. Analysis and interpretation would then only be done on a patient-specific and population-based basis as is currently done for testing and screening purposes. Perhaps even current criteria, or some evolution of them, could be used for choosing which analysis and interpretation needs to be done on whom. If this future scenario is correct, then the hereditary genetic testing market will require two services; large-scale, high-quality genome sequencing and genomic information storage followed by analysis and interpretation of said data. Genome sequencing itself will likely become a “commodity” in that it will be a service that is widely available and similar in quality among different companies. Thus, it will be difficult to have it sufficiently profitable to fund genetic counseling services as currently provided.

There are a few potential approaches by which companies could make money on the genetic analyses/interpretation component of this possible future. One is to maintain propriety (albeit non-patentable) information on genotype-phenotype correlations. This has been Myriad’s approach for maintaining market share of BRCA testing. But the increased competition in BRCA testing has caused more publicly available BRCA genotype-phenotype correlation information to be available. There has been an increase in the sharing of information among commercial and non-commercial laboratories that has decreased the amount of proprietary information (Free the Data n.d.; Guerrini et al. 2017). It is unlikely that any company will be able to maintain a proprietary position on genotype-phenotype correlations over the long-term given that the current trend is in the direction of more information sharing.

It is also conceivable that companies will develop better analytical and interpretative tools that could give them a competitive advantage. This would be akin to Google developing a better search engine that provides the desired information faster and with higher accuracy than competitors. In addition, companies could develop better interfaces for healthcare providers and patients to access the analyses and interpretations. This would be analogous to Facebook dominating the social network world through innovation of its platform and a standardization of its format that rewards repeat users. Another model could be seen in the Apple App Store approach wherein patients use specific apps to access their genomic information for various purposes (e.g., Helix’s model) (Farr 2016).

The bulk data of genetic information without personal identifiers may also be monetized due to its inherent value to biopharmaceutical companies for the development of new therapies. Genotype-phenotype correlations could provide leads for new drug targets. Purveyors of “personalized medicine” could also use this information for the aim of tailoring biopharmaceuticals for specific populations that share certain genetic markers. Genetic testing companies have been monetizing this information for many years now and this trend should be expected to continue and expand. For example, 23andMe has been selling their customers’ genetic data to biopharmaceutical companies since it was founded and has even recently begun their own biopharmaceutical development program (Herper n.d.).

## Medical Genetic Testing Companies and the Future of Genetic Counseling Support

We conjecture several potential outcomes for these medical genetic testing companies as they attempt to provide a suitable ROI to their investors and other shareholders. Each of these potential trajectories will have a varying degree of impact on genetic counselors.

Perhaps this is most clearly seen in the changing role of genetic counselors over the past decade. Because of limited support from clinics, Medicaid, Medicare, and insurance companies, many genetic testing companies directly provide pre- and post-test genetic counseling services to patients. Genetic counselors from these companies are also made available to advise physicians. With the exception of Myriad’s waning BRCA franchise legacy, most of these companies are not currently profitable and they must be funding their genetic counselor services from the monies they raise from investors. If they continue to be unprofitable or not sufficiently profitable to justify an acceptable ROI—then they will have difficulty raising additional funds. Thus, their penchant to support genetic counseling services will likely decrease.

At the same time, companies are employing genetic counselors in less direct counseling roles such as sales and marketing, report writing, and variant interpretation. There is also the risk that employment of genetic counselors for these non-counseling activities may become less supported as investor funding becomes less available.

While companies could become profitable, and stay independent from decreased testing costs and increased market size, independence does not imply a future reliance on genetic counselors. Companies could change their focus away from services that require genetic counselors. Myriad, for example, is investing more on non-hereditary testing, a pivot which will likely reduce their need for genetic counselors.

Mergers between genetic testing companies could also have varied effects on genetic counselors. For example, counseling more directly related to individual patients is likely to scale with the number of test conducted by the merged entity, whereas jobs less directly involved in counseling individual patients (e.g., marketing) may be reduced by such mergers as the new entities attempt to become more efficient.

Companies could maintain their interest in hereditary and prenatal genetic services and such services could evolve into more analytic and interpretative services (instead of the actual sequencing). This would then likely cause an increased demand for genetic counselors to perform such activities. This would include not only work in the development and implementation of such genetic information technology but also patient counseling before and after specific analytic and interpretative services are rendered (akin to pre- and post-test counseling). In this scenario, it is likely that genetic counselors would be valued for their other non-counseling activities in customer liaison and marketing and sales.

Genetic testing companies could also be bought by bigger reference laboratories. This would seem to be an attractive “exit strategy” for struggling companies given the current market and as evidence by the acquisition of Sequenom by Lab Corp and Ariosa Diagnostics by Roche Diagnostics for example. This could conceivably be the predominant plan for these independent companies as reflected in their keen interest in acquiring market share with the hope of seeming more valuable to potential buyers. It is not clear how such consolidating acts would affect genetic counseling. Some of the larger diversified companies provide pre- and post-test genetic counseling. Others refrain from any genetic counseling and adhere to a more restricted role of providing the testing with limited direct patient contact. LabCorp, for example, employs over 100 genetic counselors to provide direct patient care, while Quest Diagnostics provides neither pre- nor post-test counseling (Quest Diagnostics does employ some genetic counselors in other roles such as test reporting, product management, and as liaisons to healthcare providers). In the future, these larger testing companies could also be less inclined to provide counseling services if their genetic testing volume were more dependent on bundling genetic tests with their panoply of other non-genetic tests and less dependent on offering genetic counseling services in order to facilitate the uptake of genetic tests. Given the anticipated decreased margins of genetic testing, greater interest will be had in off-setting genetic counseling costs to other payers.

The continued transformation of healthcare systems could make genetic companies more valuable to other types of large companies. For example, Konica Minolta, a diversified, healthcare technology company, recently acquired Ambry Genetics (a privately held genetic testing company) so as to develop its precision medicine offerings. Highly profitable, electronic medical record (EMR) companies may also be inclined to acquire genetic information companies to better integrate genetic information into the wider sphere of electronic medical records (Harris n.d.). It is likely that these diversified healthcare and EMR companies will continue to invest in the development of genetic information tools which would require the help of genetic counselors. However, it is unlikely that such companies would support clinical genetic counseling services since they do not typically provide actual, direct clinical services.

Changes in healthcare policy could also affect the role of genetic counselors who work for laboratories (Pollock 2012). Some states have enacted laws which prohibit laboratory-employed genetic counselors to provide direct patient services. Additionally, some insurance plans require genetic counseling by an independent genetic counselor, one not affiliated with a testing laboratory, prior to authorizing payment for a genetic test. Increased restrictions such as these may discourage labs from employing genetic counselors for these types of roles.

## Conclusion

Genetic counselors are increasingly moving from their direct patient-care roles in clinics and hospitals to a more diversified role in commercial genetic testing companies. Several business and market forces are shaping the future role of genetic counselors in companies as these firms strive to achieve profitability and provide a suitable return-on-investment to their investors. Although not widely perceived, many commercial genetic companies are currently unprofitable. These companies have been relying on investments from outside groups to support their patient genetic counseling services and their more business-related activities of genetic counselors (e.g., marketing and sales). It is unclear whether they will be able to create sufficient profits or continue to raise enough capital to continue to support such activities. In addition, some of the genetic testing companies are likely to be bought by bigger companies which will affect the role of genetic counselors. Finally, the entire paradigm of genetic testing is likely to shift to a one-test-many analyses/interpretation model in which the one test will be WES or WGS. How this all plays out and affects the genetic counseling work force is in flux and will require genetic counselors to be highly adaptive to these changing work environments.
